# Microarray analysis of gene expression profiles in ripening pineapple fruits

**DOI:** 10.1186/1471-2229-12-240

**Published:** 2012-12-18

**Authors:** Jonni H Koia, Richard L Moyle, Jose R Botella

**Affiliations:** 1 Plant Genetic Engineering Laboratory, School of Agriculture and Food Sciences, University of Queensland, Brisbane, 4072, Australia

**Keywords:** Pineapple, Non-climacteric, Fruit ripening, Microarray

## Abstract

**Background:**

Pineapple (*Ananas comosus*) is a tropical fruit crop of significant commercial importance. Although the physiological changes that occur during pineapple fruit development have been well characterized, little is known about the molecular events that occur during the fruit ripening process. Understanding the molecular basis of pineapple fruit ripening will aid the development of new varieties via molecular breeding or genetic modification. In this study we developed a 9277 element pineapple microarray and used it to profile gene expression changes that occur during pineapple fruit ripening.

**Results:**

Microarray analyses identified 271 unique cDNAs differentially expressed at least 1.5-fold between the mature green and mature yellow stages of pineapple fruit ripening. Among these 271 sequences, 184 share significant homology with genes encoding proteins of known function, 53 share homology with genes encoding proteins of unknown function and 34 share no significant homology with any database accession. Of the 237 pineapple sequences with homologs, 160 were up-regulated and 77 were down-regulated during pineapple fruit ripening. DAVID Functional Annotation Cluster (FAC) analysis of all 237 sequences with homologs revealed confident enrichment scores for redox activity, organic acid metabolism, metalloenzyme activity, glycolysis, vitamin C biosynthesis, antioxidant activity and cysteine peptidase activity, indicating the functional significance and importance of these processes and pathways during pineapple fruit development. Quantitative real-time PCR analysis validated the microarray expression results for nine out of ten genes tested.

**Conclusions:**

This is the first report of a microarray based gene expression study undertaken in pineapple. Our bioinformatic analyses of the transcript profiles have identified a number of genes, processes and pathways with putative involvement in the pineapple fruit ripening process. This study extends our knowledge of the molecular basis of pineapple fruit ripening and non-climacteric fruit ripening in general.

## Background

Pineapple (*Ananas comosus*) is a tropical fruit crop of significant commercial value and is the most important economic plant in the *Bromeliaceae* family. However, surprisingly little research has been undertaken to understand the molecular basis of pineapple fruit development - an essential pre-requisite for future improvement by molecular breeding or genetic modification.

In terms of fruit ripening, fleshy fruits are classified as either climacteric or non-climacteric. In climacteric fruits, such as apples and bananas, a burst of ethylene biosynthesis and an increase in respiration is observed at the onset of ripening. Conversely, non-climacteric fruits such as pineapple lack the autocatalytic ethylene burst and the increase in respiration. Although the physiological changes that occur during pineapple fruit development are well known, little is known about the molecular events that govern pineapple fruit ripening.

The advent of high throughput sequencing and microarray technologies has facilitated large-scale studies on gene expression changes during fruit development in a number of species, including climacteric fruits tomato and apple, and the non-climacteric fruit strawberry [1-3]. In a previous study, we reported the construction of subtracted and normalized EST cDNA libraries, including the green mature and yellow mature ripening stages of pineapple fruits [4]. More recently, short read next generation sequencing technology was applied to a ripe pineapple fruit gene discovery project [5]. In this study, we report the development of an EST-based pineapple microarray and its use to identify differentially expressed genes during fruit ripening. Online bioinformatics tools were used to assign putative identity and function to those pineapple genes displaying differential expression during fruit ripening. We also applied visual mapping tools such as MapMan (v3.1.1), the Kyoto Encyclopedia of Genes and Genomes (KEGG) online resource and Heat Maps generated through DAVID to visualize biological processes and pathways of significance during pineapple fruit ripening. Quantitative real-time PCR analysis was used to validate a subset of the differentially expressed genes. This study contributes to our understanding of the molecular basis of pineapple fruit ripening and non-climacteric ripening in general.

## Results and discussion

### Microarray analysis of pineapple fruit ripening identifies gene expression changes associated with important metabolic pathways and processes

9277 cDNAs isolated from several pineapple tissues including roots, green mature and yellow mature fruits were spotted in duplicate onto microarray slides (Australian Genome Facility, University of Queensland). Hybridization was carried out using probes derived from mature green fruits and fully ripened yellow fruits. In total, three replicate microarray hybridizations were performed and analysis of the results identified 271 differentially expressed ESTs (> 1.5 fold). Of these, 160 were up-regulated and 77 down-regulated during pineapple fruit ripening. Among the 271 EST sequences, 184 shared significant homology with known gene products, 53 shared homology with products of unknown function, and 34 had no significant homology with any known plant gene sequence in the GenBank database. EST sequences with homologs were subjected to functional classification analysis and assigned an *Arabidopsis thaliana* homolog ID tag (e.g. At2g43640). The microarray data generated in this study has been deposited in NCBI's Gene Expression Omnibus [6] and are accessible through GEO series accession number GSE38521 (http://www.ncbi.nlm.nih.gov/geo/query/acc.cgi?acc=GSE38521).

**Figure 1:**
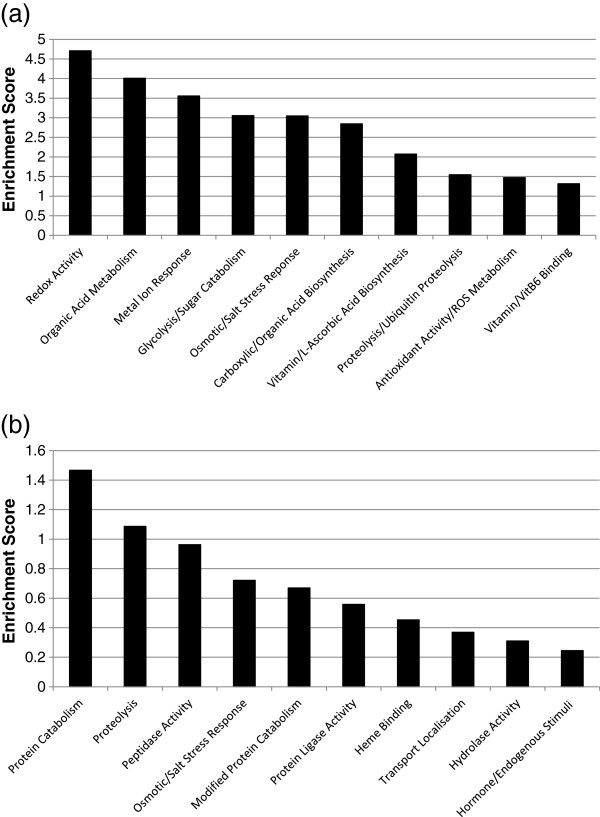
**DAVID Functional Annotation Cluster (FAC) analysis of normalized and annotated genes during pineapple fruit development.** (**a**) Major FACs for up-regulated genes (> 1.5-fold). (**b**) Major FACs for down-regulated genes (> 1.5-fold). Significance is determined by corresponding enrichment scores.

The expression data was analyzed using the Functional Annotation Cluster (FAC) tool contained in the Database for Annotation, Visualization and Integrated Discovery (DAVID) [7]. DAVID FAC analysis of the 160 up-regulated genes (> 1.5-fold) produced a total of 37 enriched functional clusters under high stringency conditions. Redox activity, organic acid metabolism, metalloenzyme activity, glycolysis, vitamin C biosynthesis and antioxidant-ROS activity showed high enrichment scores with strong confidence levels (EASE score). The enrichment score gives an indication of the biological significance of the gene groups being analyzed, from which the top 10 were considered in our study (Figure 1a). DAVID FAC analysis of the 77 down-regulated genes (> 1.5-fold produced 16 enriched functional clusters under high stringency conditions and protein catabolism was the most significant biological process in green mature fruit (Figure 1b).

### Redox activity

**Figure 2:**
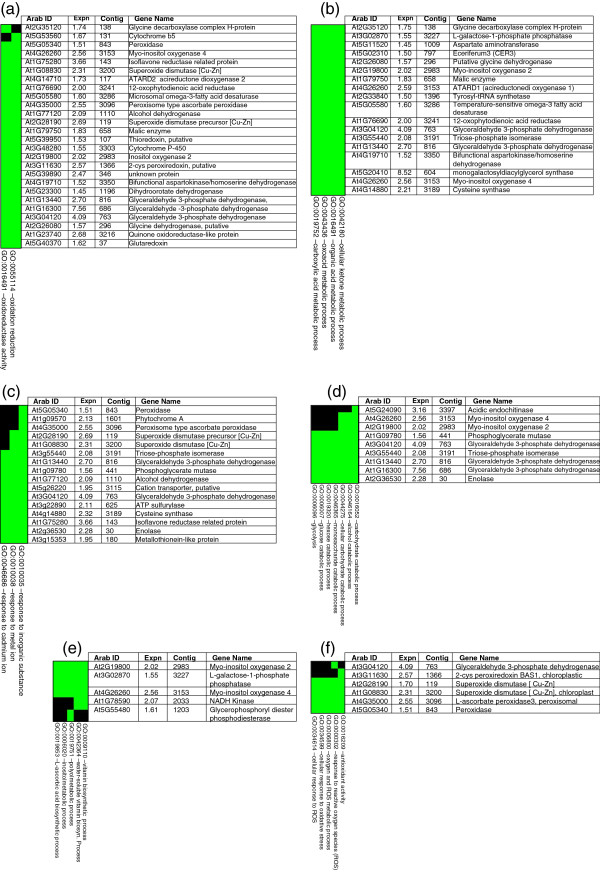
**DAVID heat map analysis of biologically significant FACs containing pineapple up-regulated genes (> 1.5-fold).** (**a**) Redox activity. (**b**) Organic acid metabolism. (**c**) Metalloenzyme activity. (**d**) Glycolysis and sugar catabolism. (**e**) Vitamin and L-ascorbate metabolism. (**f**) Antioxidant activity and ROS metabolism. Green and black shading indicates positive and unconfirmed correlation of annotated pineapple gene and functional GO terms, respectively. ‘Arab ID’ shows the closest Arabidopsis homologue. ‘Expn’ means expression fold. ‘Contig’ identifies the clone in the pineapple database (http://www.pgel.com.au).

Reduction-oxidation (redox) activity showed the highest enrichment score in the FAC analysis of genes up-regulated in mature yellow fruits (Figure 1a). DAVID heat map analyses identified 26 genes with an expression range of 1.51 to 7.56 that functionally clustered into common GO terms related to oxidation-reduction and oxidoreductase activity (Figure 2A). A number of genes identified in the heat map relate to dismutase, peroxiredoxin, glutaredoxin, ascorbate-glutathionine and thioredoxin activities.

The state of oxidation for a given organism is influenced by stress response-related enzymes such as dismutases, peroxidase, glutaredoxin, ascorbate-glutathionine and thioredoxin [8]. The state of oxidation may influence the rate at which ethylene is produced at the onset of ripening in climacteric fruits [9]. Fruit maturation may be thought of as a catabolic process involving an array of redox enzymes causing degradation of fruit tissue and emanation of ethylene as a catabolic by-product that accelerates ripening. Possible differences between the oxidation state and modes of catabolism stress responses ought to be investigated between climacteric and non-climacteric fruit species. The observed redox activity in pineapple might also be related to the large size of the fruit and the low partial pressure of oxygen.

### Organic acid metabolism

FAC analysis identified organic acid metabolism as an important biological process during pineapple fruit ripening (Figure 1a). DAVID heat map analyses identified 18 genes with an expression range of 1.50 to 8.52 that functionally clustered into common GO terms related to organic acid metabolism and other associated processes such as cellular ketone metabolism, oxoacid metabolism and carboxylic acid metabolism (Figure 2b).

Organic acid content has been reported to increase during ripening of climacteric and non-climacteric fruits [10-13]. In pineapple, acidity increases during development and starts declining once the fruit approaches maturity and ripens [14]. Citric and malic acid are the two main organic acids that contribute to the sourness and acidity of most climacteric and non-climacteric fruits such as peach [11,15,16], apple [17], kiwifruit [18], grape [19], orange [20], cherry [21], strawberry [12] and pineapple [22]. Furthermore, citric acid was shown to undergo the greatest increase in developing pineapple, reaching a peak prior to fruit ripening, whereas malic acid showed little change during development [22].

### Metalloenzyme activity

FAC analysis identified responses to inorganic compounds, metal ion cofactors and cadmium as significant biological processes during pineapple fruit ripening (Figure 1a). A total of 16 genes ranging in expression from 1.51 to 4.09 fold, were identified in the DAVID heat map (Figure 2c).

A number of metalloenzymes activated in response to inorganic substances, metal ion cofactors and cadmium were identified in the heat map analysis (Figure 2c). These include superoxide dismutase (SOD), metallothionein (MET) and phytochrome A (PHYTA) homologs. Metalloenzymes are known to regulate the redox state of many plant crops and are involved in many important biological processes such as oxidative stress, metal ion homeostasis, pathogenicity, metal-mediated catalysis and cell death. Pineapple waste (over-ripened fruit) has been used in the bioremediation removal of toxic heavy metals from wastewater and sewage plants [23,24]. This is possibly due to an abundance of metalloenzymes such as MET, SOD and PHYT in ripe fruit, where these enzymes have the ability to act as ligands and chelate excess metal ions in wastewater treatment procedures. Indeed, MET has been implicated in cadmium detoxification and metal ion homeostasis [25]. Previous studies confirm that yellow pineapple fruit contains a small transcriptome that is dominated by high levels of metallothionein transcript [4].

### Glycolysis and sugar catabolism

**Figure 3:**
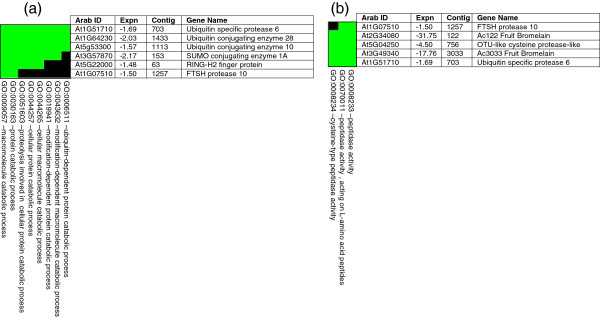
**DAVID heat map analysis of biologically significant FACs containing pineapple down-regulated genes (> 1.5-fold).** (**a**) Proteolysis and ubiquitin-dependent catabolism. (**b**) Cysteine-type peptidase activity. Green and black shading indicates positive and unconfirmed correlation of annotated pineapple gene and functional GO terms, respectively. ‘Arab ID’ shows the closest Arabidopsis homologue. ‘Expn’ means expression fold. ‘Contig’ identifies the clone in the pineapple database (http://www.pgel.com.au).

FAC and DAVID heat map analysis identified genes involved in glycolysis and sugar catabolism (Figure 1a, Figure 2d). KEGG pathway visual analysis identified six genes in the glycolysis pathway map that were up-regulated in yellow ripe fruits by 1.56 to 7.56 fold (Figure 3a, Figure 2d, Table 1). These findings indicate the importance of glycolysis and sugar catabolism during pineapple fruit development. Most of the genes identified on the KEGG map were also identified in the corresponding heat map matrix.

**Table 1:** **List of pineapple ESTs from KEGG pathway maps with contig ID numbers (**http://www.pgel.com.au**), *Arabidopsis *ID tag, normalized expression value and annotated gene description**

**Schematic Name**	**Arabidopsis homologue CloneID**	**Normalized Expression Levels**	**Description**	**KEGG** **ID**
**Glycolysis - KEGG PATHWAY**				
contig 763	At3g04120	4.1	Glyceraldehyde 3-phosphate dehydrogenase, cytosolic	Ac763
contig 251	At4g33070	3.4	Pyruvate decarboxylase 1	Ac251
contig 30	At2g36530	2.3	Enolase	Ac30
contig 1110	At1g77120	2.1	Alcohol dehydrogenase	Ac1110
contig 3191	At3g55440	2.1	Triose-phosphate isomerase	Ac3191
contig 441	At1g09780	1.6	Phosphoglycerate mutase	Ac441
**Cysteine and Methionine Metabolism**				
contig 3189	At4g14880	2.3	Cysteine synthase	Ac3189
contig 117	At4g14710	1.7	ATARD2 acireductone dioxygenase2	Ac117
contig 113	At3g25570	1.6	S-adenosylmethionine decarboxylase	Ac113
contig 1009	At5g11520	1.5	Aspartate aminotransferase	Ac1009
contig 3350	At4g19710	1.5	Bifunctional aspartokinase/homoserine dehydrogenase	Ac3350
**Ubiquitin-Mediated Proteolysis - KEGG Pathway**				
contig 1299	At2g02760	3.1	Ubiquitin conjugating protein	UBE2A
contig 154	At1g16890	1.6	Ubiquitin carrier protein E2 36	UBE2N
contig 861	At5g22920	1.6	Putative PGPD14 protein (pollen germination related protein)	PIRH2
contig 1793	At5g42190	1.5	SKP1-like protein 1B	SKP1
contig 1433	At1g64230	−2.0	Ubiquitin carrier protein E2 28	UBE2D_E
contig 153	At3g57870	−2.2	Ubiquitin carrier protein	UBE2I

Interestingly, gene expression profiling of non-climacteric grape, also identified a significant cluster of up-regulated genes involved in glycolysis and sugar catabolic activity [26]. Taken together, the results in grape and pineapple underlay the significance of glycolytic activity during non-climacteric ripening. It is possible that ripening fruits generate increased ATP energy reserves and reducing power by increasing glycolysis that could be later used by enzymes involved in important fruit metabolic processes. The up-regulation of glycolysis related genes might also be related to phloem unloading of sugars and their storage in the vacuole.

### Anti-oxidant activity and reactive oxygen species (ROS) metabolism

FAC analysis shows a significant enrichment of L-ascorbate (vitamin C) biosynthesis genes, anti-oxidant activity and ROS metabolism during pineapple fruit ripening (Figure 1a). DAVID heat map analysis revealed a total of five pineapple genes that functionally cluster with GO terms related to vitamin biosynthesis, polyol metabolism, inositol metabolism and L-ascorbic acid biosynthesis (Figure 2e). To name a few, myo-inositol oxygenase 4 (MIOX4), myo-inositol oxygenase 2 (MIOX2) and L-galactose-1-phosphate phosphatase (GPP), were identified with expression levels ranging from 1.55 to 2.56-fold increase. Radiotracer evidence showed that grape GPP (VTC4) is a key enzyme involved in L-ascorbate biosynthesis as non-climacteric grape fruits develop [27]. Our pineapple GPP expression profile also correlates with the peach [28] and tomato [29] GPP orthologs during fruit ripening. The pineapple MIOX4 and MIOX2 genes correlate with MIOX orthologous genes found in grape [30] and tomato [29] and are involved in the conversion of myo-inositol to D-glucoronic acid during L-ascorbic acid biosynthesis.

DAVID heat map analyses also identified a cluster of six pineapple genes found to be involved in anti-oxidant activity, response to ROS, oxygen and ROS metabolism, cellular response to oxidative stress and cellular response to ROS activity (Figure 2f). L-ascorbate [27,31] and glutathione [32,33] have been linked to plant antioxidant activity and fruit ripening. In fact, fruit ripening is considered to be an oxidative process, where L-ascorbate and glutathione are linked by a cycle of enzymes whose biological role is to detoxify hydrogen peroxide [34]. KEGG pathway mapping (Figure 3b, Table 1) identified a cluster of genes involved in L-cysteine biosynthesis, an important precursor for glutathione. Aside from ripening, L-ascorbate production is influenced by various environmental cues such as light [35], temperature [29,36] and ambient ozone concentrations [37]. These stresses induce the formation of ROS, which are removed by the plant’s antioxidant system involving catalase, superoxide dismutase, peroxidases and enzymes involved in the ascorbate-glutathione cycle [27]. Non-climacteric grape orthologs of pineapple GPP, APOX3, PXDN-B1, SOD-*119* and SOD-*3200* were also reported to be involved in antioxidant activity [30]. Hydrogen peroxide (H_2_O_2_) is the most stable ROS species and plays a crucial role as a signaling molecule in various physiological processes [38]. H_2_O_2_ is also involved in cell membrane and wall degradation of fleshy tomato fruit tissue, thereby causing fruit softening [39]. This may also be the case in developing pineapple fruit tissue.

### Proteolysis and Ubiquitin-type housekeeping processes

**Figure 4:**
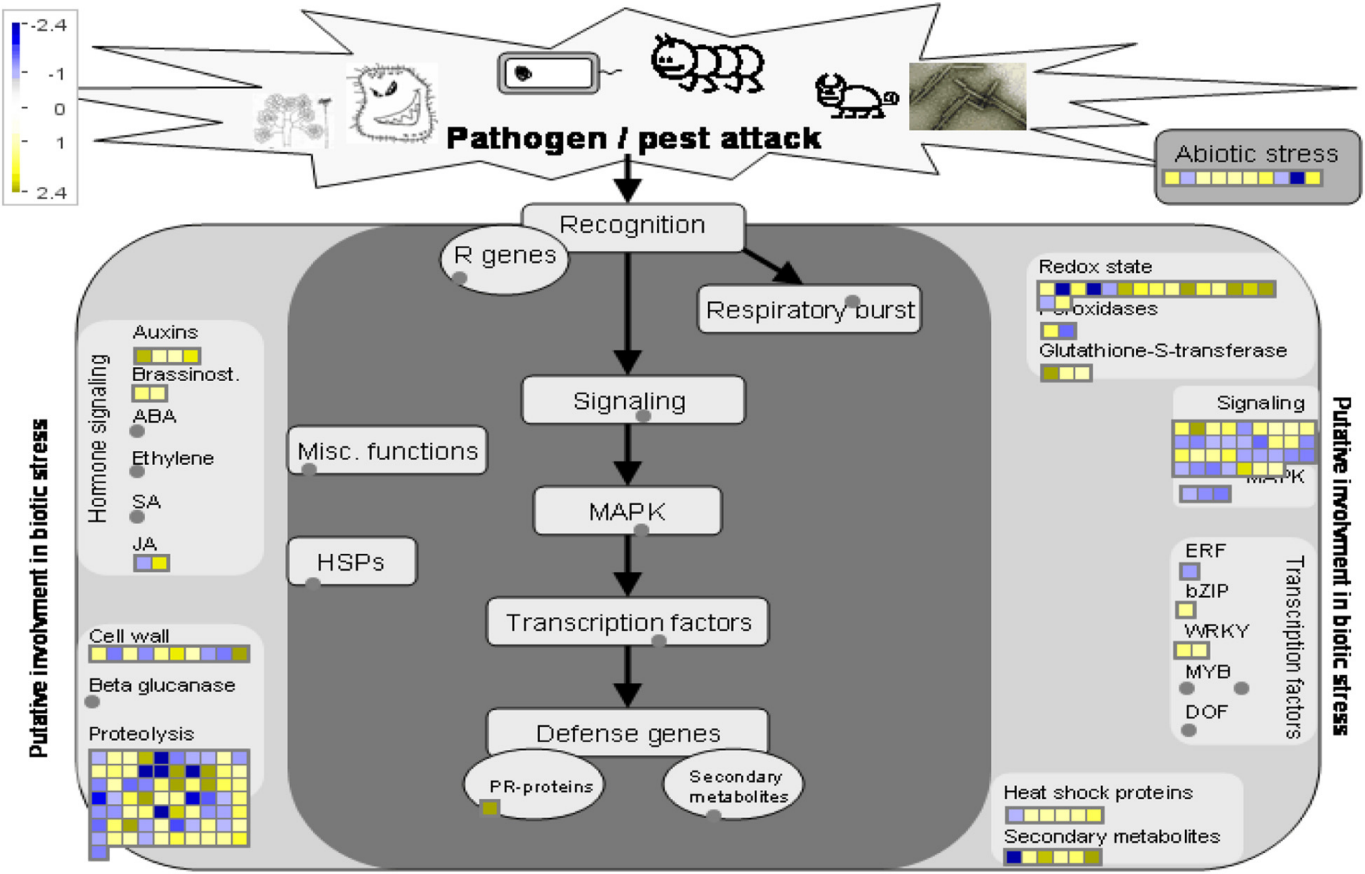
**MapMan (3.1.1) pathway analysis of differentially expressed genes (> 1.1-fold). Biotic and abiotic stress overview.** Yellow and blue boxes indicate up-regulated and down-regulated homologs, respectively. Expression value scale is provided. See Table 2 for more detail.

**Figure 5:**
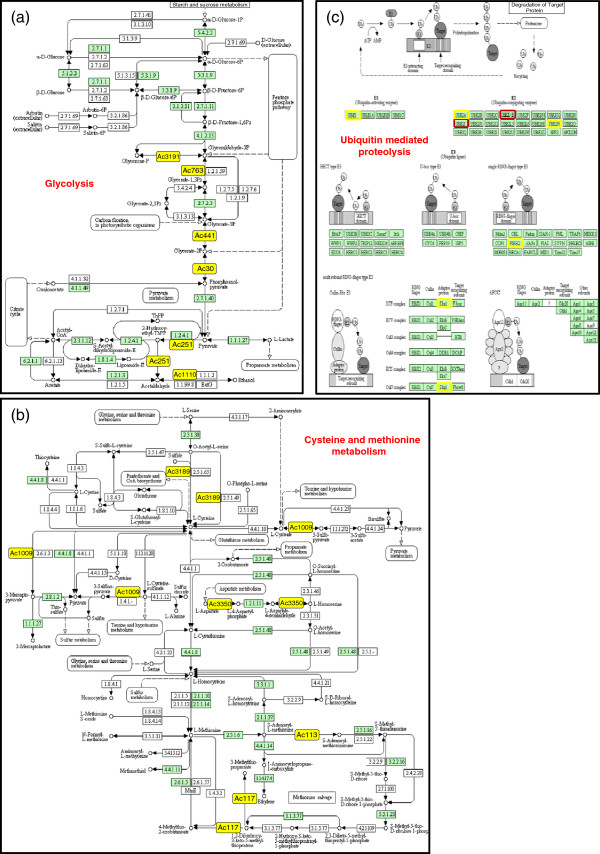
**KEGG pathway map analyses of differentially expressed genes (> 1.5-fold).** (**a**) Glycolysis. (**b**) Cysteine and methionine metabolism. (**c**) Ubiquitin mediated proteolysis. Yellow and red boxes (filled and bordered) indicate up-regulated and down-regulated homologs, respectively. All pineapple homologs identified on the KEGG maps are shown in Table 1.

DAVID FAC analysis indicated that proteolysis and ubiquitin protein catabolism are active in both green and yellow pineapple fruit (Figure 1a, 1b). The enrichment score for proteolysis and ubiquitin protein catabolism was comparable between both FAC analyses (Figure 1a, 1b), however it was significantly lower than other up-regulated biological processes (Figure 1a). DAVID heat map analysis identified six down-regulated genes (Figure 4a) and 14 up-regulated genes (not shown) involved in proteolysis and ubiquitin protein catabolism. KEGG pathway analysis visually created an ubiquitin mediated proteolysis map (Figure 3c, Table 1), showing genes which are active in both yellow (up-regulated) and green (down-regulated) mature pineapple fruit. MapMan (v3.1.1) pathway analysis of a biotic-abiotic overview (Figure 5, Table 2) also identified 30 down-regulated genes and 41 up-regulated genes involved in proteolysis and ubiquitin-type activity during pineapple fruit ripening.

**Table 2:** **List of pineapple ESTs from MapMan maps with contig ID numbers (**http://www.pgel.com.au**), *Arabidopsis *ID tag, normalized expression value and annotated gene description**

**Schematic Name**	**Arabidopsis homologue CloneID**	**Normalized Expression Levels**	**MapMan Identifier**
**REDOX State-Peroxidase-Antioxidant Activity - Stress Overview**			
contig 422	At2g16060	12.2	AHB1 (ARABIDOPSIS HEMOGLOBIN 1)
contig 119	At2g28190	2.7	CSD2 (COPPER/ZINC SUPEROXIDE DISMUTASE 2)
contig 1366	At3g11630	2.5	2-cys peroxiredoxin, chloroplast (BAS1)
contig 3096	At4g35000	2.3	APX3 (ASCORBATE PEROXIDASE 3)
contig 3200	At1g08830	2.2	CSD1 (copper/zinc superoxide dismutase 1)
contig 37	At5g40370	1.6	GRXC2 (Glutaredoxin-C2)
contig 3227	At3g02870	1.5	VTC4 (2-bisphosphate nucleotidase)
contig 107	at5g39950	1.5	ATTRX2 (Arabidopsis thioredoxin h2)
**Proteolysis Activity - Stress Overview**			
contig 122	At2g34080	−31.7	Fruit bromelain
contig 3033	At3g49340	−17.8	Fruit bromelain
contig 1322	At3g45630	−5.6	RNA recognition motif (RRM)
contig 3424	At2g43210	−5.2	UBX domain-containing protein
contig 756	At5g04250	−4.5	OTU-like cysteine protease family protein

Proteolysis is essential for many aspects of plant development. It is responsible for cellular housekeeping and stress responses by removing abnormal and miss-folded proteins, supplying amino acids needed to make new proteins, controlling homeostasis by reducing the abundance of key enzymes and regulatory proteins, and controlling gene function and programmed cell death of specific plant organs or cells [40-42]. Our bioinformatics analysis indicates that protein degradation during pineapple fruit development is a complex process involving a multitude of proteolytic pathways. Selective proteolysis of damaged proteins is mainly controlled through ubiquitin-mediated processes involving ubiquitin and several ubiquitin conjugating enzymes [43].

### Pineapple fruit bromelain

DAVID FAC analysis of the 77 down-regulated genes (> 1.5-fold), suggested that cysteine type peptidase activity was the most significant process in green mature fruit (Figure 1b). This is mainly due to two pineapple fruit bromelains, Ac-*122* (−17.76-fold) and Ac-*3033* (−31.75-fold) that were identified on a DAVID heat map (Figure 4A) and found to be strongly down-regulated as pineapple ripens from mature green to mature yellow fruit. Bromelain is a cysteine-type protease unique to pineapple. Cysteine-type proteases are also found in other fruits, such as kiwifruit [44], papaya and fig [45]. Pineapple EST library analyses identified an abundance of a bromelain inhibitor (Ac124) and a fruit bromelain (Ac122) from green mature fruit [4]. The bromelain inhibitor Ac124 was the most abundant EST in the green fruit library with 52 clones isolated. Northern analyses further confirmed decreased expression of Ac122 and Ac124 as pineapple fruit develops [4]. The Ac124 bromelain inhibitor corresponds to a non-cystatin bromelain inhibitor precursor homolog in pineapple [46,47], that does not have an inhibitory effect on pineapple fruit bromelain. It is possible that Ac-*122* and Ac-*3033* are more abundant in green mature pineapple fruit due to the role that cysteine-type proteases play in plant defense mechanisms [45], particularly before fruit maturation.

### Validation of microarray data by real-time RT-PCR

**Figure 6:**
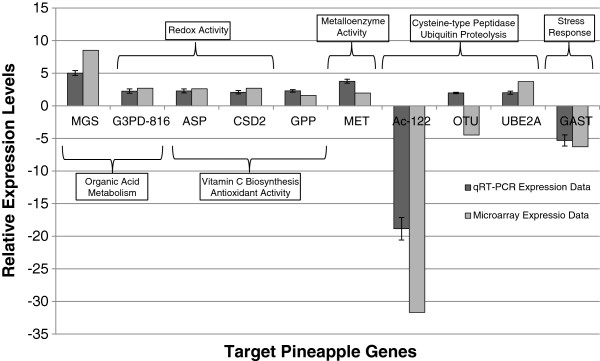
**Validation of microarray data.** A total of ten pineapple genes showing differential expression in our microarray experiments were randomly selected and their relative expression determined using qRT-PCR. See Table 3 for more detail.

In order to validate our microarray results we performed quantitative real time PCR (qRT-PCR) to determine the expression levels of ten pineapple genes randomly selected from the list of genes differentially expressed across mature green and mature yellow fruits. To avoid bias, the tested genes were chosen from an array of different processes including redox activity, organic acid metabolism, metalloenzyme activity, vitamin C biosynthesis, antioxidant activity, cysteine-type peptidase activity and ubiquitin proteolysis. The qRT-PCR expression results correlated with the microarray expression data for 9 out of the 10 genes tested (Figure 6, Table 3). qRT-PCR determination of MGS and G3PD-*816* mRNA levels showed a 2.6-fold and 5.0-fold increase respectively in mature yellow fruits over mature green fruits and these results compare favorably to the 2.7-fold and 8.5-fold increase in expression determined by the microarray analysis. Both of these genes are involved in organic acid metabolism. ASP, GPP and CSD2 are involved in vitamin C biosynthesis, redox and antioxidant activity. qRT-PCR analysis showed an increase of 2.6-fold, 2.1-fold and 2.3-fold in the levels of ASP, GPP and CSD2 respectively. These results are in good agreement with our microarray data that showed 2.3-fold (ASP), 1.5-fold (GPP) and 2.7-fold (CSD2) increases in expression during fruit ripening (Table 3). Expression levels of genes involved in cysteine-type peptidase (Ac-*122*) and ubiquitin proteolysis (OTU and UBE2A) were also quantitatively analyzed. Real-time data confirmed that Ac-*122* is significantly down-regulated (20.6-fold) and UBE2A up-regulated (2.1-fold) as pineapple fruit ripens. Our microarray data correlated with the qRT-PCR data, showing a 31.7-fold down-regulation of Ac-*122* and 3.1-fold up-regulation of UBE2A. Expression analysis of OTU by qRT-PCR revealed a 2.0-fold up-regulation, in contrast with the microarray data which showed OTU to be down-regulated by 4.5-fold. OTU belongs to the cysteine proteinase superfamily [48], a large multigene family that is likely to contain genes with highly similar sequences. While qRT-PCR results are usually accurate and gene specific, the microarray used in this study was constructed spotting cDNA clones and it is therefore possible that the hybridization results for genes encoded by multigene families could be adulterated by cross-hybridization of homologous probes. qRT-PCR analysis further confirmed up-regulated expression of MET (3.5-fold) and down-regulated expression of GAST (4.5-fold), involved in metalloenzyme activity and stress response pathways respectively. The microarray data correlated with qRT-PCR showing a 1.95-fold (MET) increase and 6.3-fold (GAST) decrease, respectively. MET cDNA levels were found to be highly abundant in yellow pineapple fruit tissue and shown to be significantly up-regulated during pineapple fruit development [4].

**Table 3:** qRT PCR validation of microarray results

**Schematic Name**	**Microarray Result**	**qRT-PCR-Result**	**Target Gene**
**Redox Activity and Metalloenzyme Activity**			
contig 816	2.7 increase	2.6 increase	GSPD-*816* (glyceraldehyde 3-phosphate dehydrogenase, cytosolic)
contig 3096	2.3 increase	2.6 increase	ASP (peroxisome type ascorbate peroxidase)
contig 119	2.7 increase	2.3 increase	CSD2 (copper/zinc dismutase 2)
**Metalloenzyme Activity**			
contig 180	1.95 increase	3.5 increase	MET (metallothionein)
**Vitamin C Biosynthesis and Antioxidant Activity**			
contig 3096	2.3 increase	2.6 increase	ASP (ascorbate peroxidase, peroxisome type)
contig 119	2.7 increase	2.3 increase	CSD2 (copper/zinc dismutase 2)
contig 3227	1.5 increase	2.1 increase	GPP (L-galactose-1-phosphate phosphatase)
**Organic Acid Metabolism**			
contig 816	2.7 increase	2.6 increase	GSPD-*816* (glyceraldehyde 3-phosphate dehydrogenase, cytosolic)
contig 604	8.5 increase	5.0 increase	MGS (monogalactosyldiacylglycerol synthase)
**Stress Related**			
contig 35	6.3 decrease	4.5 decrease	GAST (GAST-like protein)
**Cysteine-Type Peptidase and Ubiquitin Proteolysis**			
contig 122	31.7 decrease	20.6 decrease	Ac-*122* (Fruit Bromelain Ac-*122*)
contig 756	4.5 decrease	2.0 increase	OTU (OTU-like cysteine protease family protein)
contig 1299	3.1 increase	2.1 increase	UBE2A (Ubiquitin conjugating protein)

## Conclusions

In this study we have analyzed the changes in expression levels during pineapple fruit ripening using a purpose built pineapple microarray containing 9,277 EST elements. We identified 271 unique pineapple clones with differential expression of at least 1.5-fold from mature green to mature yellow stages of fruit ripening. Of these 271 ESTs, 237 displayed significant homology to plant sequences encoding proteins of known or unknown function. Of these, 160 ESTs were up-regulated and 77 ESTs down-regulated during pineapple fruit ripening. Furthermore, quantitative real-time PCR analysis validated the microarray results for nine out of ten genes randomly chosen from different cellular processes.

Bioinformatics analyses of the microarray results suggest that redox activity is a prominent biological process during pineapple fruit ripening. Organic acid metabolism of citric and malic acid and metalloenzyme activity were also up-regulated during pineapple fruit ripening. The significant increases observed in metalloenzyme encoding genes, may contribute to the bioremediation properties of pineapple waste [23]. Glycolysis related genes were also significantly up-regulated during pineapple fruit ripening, possibly to generate ATP energy reserves for important ripening processes or in response to the influx of sugars and their storage in the vacuole.

Furthermore, L-ascorbate biosynthesis, antioxidant activity and ROS metabolism were determined to be up-regulated during pineapple fruit development. The antioxidant properties of L-ascorbate and glutathionine may be involved in detoxifying H_2_O_2_ as pineapple fruit ripens. H_2_O_2_ may have a biological role activating an up-regulated response of vitamin C accumulation and antioxidant activity during pineapple fruit development.

Our studies also identified two pineapple bromelains, Ac-*122* and Ac-*3033*, which were highly abundant in green mature pineapple tissues and may possibly be involved in plant defense mechanisms against pathogenic attack prior to fruit maturation.

Applying a microarray based approach to studying large scale gene expression changes during fruit development has contributed to our understanding of the molecular basis of pineapple fruit ripening and non-climacteric fruit ripening in general. The development of the pineapple microarray will also enable the future large scale analysis of gene expression changes in other pineapple developmental processes, conditions and treatments.

## Methods

### Plant material and RNA extraction

Commercial field-grown pineapples (*A. comosus* L. Smooth Cayenne, Clone C10) were harvested early in the morning and graded according to ripening appearance [4]. The skin and pith tissues were removed and the middle third of the fruit flesh was snap frozen in liquid nitrogen, pulverized, and stored at −80°C. RNA was extracted using TRIzol reagent (Invitrogen).

### Microarray design and transcript profiling

PCR amplified cDNA elements derived from pineapple fruit, root tip, root and nematode infected roots and gall cDNA libraries were printed in duplicate onto Corning UltraGAPS slides by the Microarray Facility at the ARC Special Research Centre for Functional and Applied Genomics, University of Queensland [49]. The array contains 5533 previously sequenced cDNA elements [50] and 3744 unsequenced elements.

Fruit flesh from the middle third of unripe mature green pineapple and ripe mature yellow pineapple was used for RNA extraction, as previously described [4]. The RNA was labeled with Cy3/Cy5 fluorescent dyes using the LabelStar Array Kit (Qiagen) according to the manufacturer instructions. Labeled cDNA was mixed with 20xSSC (20 μl), liquid block (12 μ), 2%SDS (4 μl) and dH_2_O (up to 200 μl total volume). Denatured labeled target was hybridized to a pineapple microarray slide in submersible hybridization cassette chambers (Ambion) at 55°C for 12 hours. The arrays were then sequentially washed in 2x SSC/0.5% SDS, 1xSSC, 0.5xSSC and 0.05xSSC for 5 minutes at room temperature. Each slide was scanned using an ArrayWorx scanner (Applied Precision) and spot-edited using SoftWorx tracker software (Applied Biosystems). Each array dataset was then imported into GeneSpring software for analysis.

For each replicate slide, the mean and background intensity values from each channel (W595, W685) were log2 transformed and normalized using the LOWESS algorithm to remove intensity dependent effects within the calculated values. Normalized values were used to calculate the Cy3/Cy5 fluorescence ratios from experimental and biological repeats before all replicates were combined. Normalized data with a p-value of < 0.1 that passed a 2-fold standard deviation (2xSD) test from each replicate slide was individually tested. All normalized data possessing a differential expression value of 1.5-fold or more was further considered (Additional file 1: Table S1). Due to redundancy features, unsequenced clones that passed the p-value and 2xSD test, and found to be differentially expressed by 1.5-fold or more were sequenced and updated in the raw data file of each slide. Data was compiled from three hybridizations, including one dye-swap replicate. The microarray data has been entered into the Gene Expression Omnibus at NCBI and the project has been assigned the accession code GSE38521.

### Real-time PCR validation

A total of ten differentially expressed genes were selected based on their function and involvement in pathways and processes important to pineapple fruit development. Biological processes such as redox activity, organic acid metabolism, metalloenzyme activity, L-ascorbate (vitamin C) biosynthesis, antioxidant activity, cysteine-type peptidase activity and ubiquitin proteolysis were considered. A total of 18 green mature pineapple fruits and 18 yellow mature pineapple fruits, were harvested in the morning and processed as previously described [4]. Three biological replicate samples were produced for both green mature and yellow mature pineapple fruit types based on six fruit per replicate. All fruit were cored, sliced from the middle third and diced prior to liquid nitrogen treatment. Frozen diced fruit samples were pooled together according to each biological replicate and tissue type prior to being ground to a fine powder under liquid nitrogen conditions. All ground tissue from each biological replicate was homogenized and thoroughly mixed prior to RNA extraction procedures. A total of 2.5ug RNA was extracted for each biological triplicate tissue type according to methods previously described [51]. The total RNA was used for RT-cDNA synthesis using SuperScript® III Reverse Transcriptase (Invitrogen, Carlsbad, CA) and the resulting cDNA template was diluted five-fold in molecular grade water (Promega, Madisson, WI). Real-time assays were conducted for all ten target genes by using the 7900HT Sequence Detection System (Applied Biosystems, Foster City, CA) and *ActinB* was used as the reference gene. Real-time assays were based on three biological and technical replicates of green mature and yellow mature pineapple fruit tissue types. Gene expression and statistical analysis was performed using SDS version 2.2.2 software (Applied Biosystems, Foster City, CA).

### Bioinformatics analysis of normalized microarray data

The National Centre Bioinformatics Information (NCBI) BlastX tool was used to putatively annotate all normalized genes (1.5-fold) of which *Arabidopsis thaliana* homolog tags were assigned to each clone using The Arabidopsis Information Resource (TAIR) online database. Gene Ontology (GO) classification systems was used to assign putative function to each clone by way of biological process, molecular function and cellular components. The Database for Annotation, Visualization and Integrated Discovery (DAVID) v6.7b [7] was used to determine pathways and processes of major biological significance and importance through the Functional Annotation Cluster (FAC) tool based on the GO annotation function.

### DAVID functional annotation cluster analysis

DAVID FAC analysis was conducted on two independent normalized gene lists containing the 1.5-fold up-regulated normalized genes and 1.5-fold down-regulated normalized genes. High stringency ease score parameters were selected, to indicate confident enrichment scores of functional significance and importance of the given pathways and processes investigated. The Gene Ontology (GO) system in DAVID was utilized to identify enriched biological themes in both gene lists.

### Mapping and visual pathway analysis

MapMan (v3.1.1) and *Kyoto Encyclopedia of Genes and Genomes* (KEGG) pathway tools were used to visually map cluster of pineapple genes involved in common pathways and processes for both pathway-specific and molecular overview purposes. KEGG pathway tools were utilized through DAVID online tools. Since visual mapping was the primary objective, all normalized genes that were differentially expressed by 1.5-fold were considered for the MapMan and KEGG pathway analysis. Heat map analyses were also conducted through DAVID to produce a matrix of enriched GO terms with common pineapple genes that were 1.5-fold or more up- and down-regulated. The green and black shading on the heat map matrix indicates a positive and negative correlation between the enriched GO term and given pineapple gene, respectively.

### Enzyme abbreviations

superoxide dismutase (SOD-*119*, SOD-*3200*), metallothionein (MET), phytochromeA (PHYTA), myo-inositol oxygenase 4 (MIOX4), myo-inositol oxygenase 2 (MIOX2), L-galactose-1-phosphate phosphatase (GPP), 2-cys peroxiredoxin BAS1 (PXDN-B1), superoxide dismutase (SOD-*119*, SOD-*3200*) and L-ascorbate peroxidase 3 (APOX3), fruit bromelains (Ac-*122*, Ac-*3033*), MGS (mono-galactosyldiacyl-glycerol synthase), glyceraldehyde 3-phosphate dehydrogenase (G3PD-*816)*, ascorbate peroxidase (ASP), copper/zinc superoxide dismutase 2 (CSD2), OTU-like cysteine protease family protein (OTU) ubiquitin conjugating protein (UBE2A), GAST-like protein (GAST).

note: where an enzyme abbreviation appears with an italicized number separated with a hyphen, it indicates an internal identifier corresponding to the related contig number for that enzyme (e.g. G3PD-*763,* SOD-*119,* Ac-*122*).

## Competing interests

The authors declare that they have no competing interests.

## Authors’ contributions

JHK undertook bioinformatics analysis of normalized microarray data, DAVID functional annotation cluster analysis, mapping and visual pathway analysis and real-time PCR validation analyses and contributed to manuscript preparation. RLM contributed to experimental design, undertook cDNA library construction, target labeling and microarray hybridizations, spot editing and normalization procedures. JRB conceived, designed and directed the research and contributed to manuscript preparation. All authors read and approved the final manuscript.

## Supplementary Material

Additional file 1 **Table S1.** Full list of microarray elements displaying at least 1.5x change in expression between mature green and mature yellow stages of pineapple fruit ripening. Systematic ID refers to the contig number assigned during EST sequence assembly. Normalized integer refers to the fold difference in expression between mature green (Pine3) and mature yellow (Pine9) stages of fruit ripening. A positive normalized integer indicates up-regulation in mature yellow fruit (or down-regulation in mature green fruit) while a negative normalized integer indicates up-regulation in mature green fruit (or down-regulation in mature yellow fruit). Click here for file
